# Dual-Retarder Mueller Polarimetry System for Extraction of Optical Properties of Serum Albumin Protein Media

**DOI:** 10.3390/s21103442

**Published:** 2021-05-15

**Authors:** Quoc-Hung Phan, Chien-Yuan Han, Chi-Hsiang Lien, Thi-Thu-Hien Pham

**Affiliations:** 1 Department of Mechanical Engineering, National United University, Miaoli 36063, Taiwan; chlien33@nuu.edu.tw; 2 Department of Electro-Optical Engineering, National United University, Miaoli 36063, Taiwan; cyhan@gm.nuu.edu.tw; 3 Department of Biomedical Engineering, International University, Ho Chi Minh City 700000, Vietnam; ptthien@hcmiu.edu.vn

**Keywords:** Mueller matrix polarimetry, surface plasmon resonance, albumin, glucose concentration

## Abstract

A dual liquid-crystal variable retarder Mueller polarimetry system incorporating a gold-based surface plasmon resonance prism coupler was proposed for extracting the optical properties of serum albumin protein media in the reflectance configuration. The feasibility of the proposed system was demonstrated by measuring the circular dichroism and circular birefringence properties of glucose tissue phantom solutions with different albumin concentrations. The results showed that the circular dichroism increased with albumin concentration, while the optical rotation angle increased with glucose concentration. Both properties reduced over time as a result of the protein glycation effect, which led to a gradual reduction in the glucose content of the sample.

## 1. Introduction

Diabetes mellitus affects an estimated 425 million people worldwide, and it is responsible for around 3.2 to 5.0 million deaths annually and a global health expenditure of approximately USD 727 billion [1]. The term “diabetes” actually covers a group of metabolic disorders characterized by high blood glucose levels over a period of time, and it is the result of the pancreas producing insufficient insulin (type 1), or the cells of the body not responding properly to the insulin produced (type 2). Effective control of the blood glucose level is essential for diabetes management, and it is most commonly performed by finger-prick testing on a daily basis. However, the invasive nature of this test is painful, costly, and there are chances of cross infection. The methods for developing noninvasive (NI) blood glucose monitoring devices have increased in recent years. Many techniques that have been proposed in the literature include optical polarimetry [2], surface plasmon resonance (SPR) [3], and Raman spectroscopy (SERS) [4]. In addition, several NI glucose monitoring devices have been commercialized, including the GlucoWatch^®^ from Cygnus Inc., with over 94% of the readings falling within the clinically acceptable A + B region of the Clarke Error Grid [5]; the TensorTip Combo Glucometer from Cnoga Medical Ltd. with a measurement accuracy of 14.4 mg/dL [6], and the FreeStyle Libre Flash system from Abbot Inc. with an accuracy of 12–21 mg/dL [7]. However, the measurement performance of these systems is still sub-optimal as they fail to take account of the glycoxidation effect, which takes place in the presence of albumin, intralipid, and D-Glucose [8,9]. Stark et al. [10] extracted the glucose concentration in protein-containing media with a resolution of 16 mg/dL. However, the albumin concentration in the measured media was limited to 1000 mg/dL, which is far lower than the actual concentration of albumin in human blood plasma (~4000 mg/dL) [11]. Thus, methods for performing glucose extraction in the presence of higher albumin concentrations and the glycoxidation effect are still required to improve the reliability of NI glucose monitoring systems.

Mueller matrix polarimetry is a well-established technique for analyzing the anisotropic properties of turbid media, particularly biological tissues [12,13,14] or glucose sensing [15]. Lu and Chipmen [16] proposed the decomposition formalism to determine the diattenuation, retardance, and depolarization of an arbitrary Mueller matrix. Qi et al. [17] extended the Lu–Chipman decomposition Mueller matrix method for turbid media in reflection geometry. Pham et al. [18] employed the decomposition Mueller matrix for extracting effective parameters of anisotropic material. However, the Mueller matrix decomposition method required a strict sequential order of matrix components. Azzam [19] proposed a differential Mueller matrix to resolve the sequential ordering. Quijano and Diego proposed a differential Mueller matrix for characterizing the optical properties of anisotropic material [20] and for reflectance and backscattering measurements [21]. Liao and Lo [22] extracted the anisotropic parameters by using the differential Mueller matrix method. The polarimetry measurement systems generally consist of a polarization state generator (PSG) and a polarization state analyzer (PSA). Many optical components have been proposed for the construction of high-accuracy PSGs, including rotated quarter-wave plates [23], photoelastic modulators (PEM) [24], electro-optic (EO) modulators [25], and liquid crystal variable retarders (LCVRs) [26,27]. LCVRs are cheaper than both PEMs and EO modulators and are more accurate than rotated quarter-wave plates. Thus, many Mueller polarimetry systems based on LVCRs have been proposed in the literature. For example, Martino et al. [28] proposed a technique for optimizing the Mueller matrix extraction results by using dual LCVRs for both the PSG system and the PSA system. Boulesteix et al. [29] used a similar system to measure the Mueller matrix of stained collagen samples. Han et al. [30] proposed a rapid full Mueller imaging polarimetry system based on LCVRs for extracting the Mueller matrix of shrimp shells under heating treatment. In previous studies [31,32], the present group proposed an enhanced Mueller polarimetry system for NI glucose concentration measurement incorporating two EO modulators and a SPR prism coupler. However, the proposed system is expensive and requires a complicated calibration of the EO components. Furthermore, the feasibility of the proposed system was evaluated using only pure tissue phantom solutions without albumin. Accordingly, the present study proposed a cheaper Mueller polarimetry system based on dual LVCRs for the extraction of the glucose concentration in samples containing albumin with a concentration as high as 1000–3000 mg/dL.

## 2. Differential Mueller Matrix Formalism for Extracting Circular Birefringence/Circular Dichroism (CB/CD) Properties

**Figure 1:**
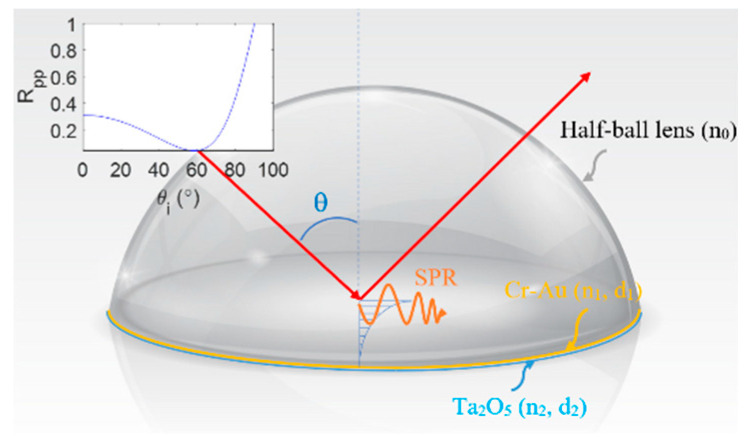
Schematic illustration of SPR prism coupler.

Figure 1 presents a schematic illustration of the SPR prism coupler incorporated within the proposed Mueller polarimetry system. As shown, the coupler has the form of a B270 glass half-ball lens (Thorlabs ACL1210U) with a Cr-Au thin film layer (thickness *d*_1_ = 20 nm) and Ta_2_O_5_ thin film layer (thickness *d*_2_ = 12 nm) coated on its lower surface. The lens and Cr-Au layer have refractive indices of 1.52 and 0.36–2.9 i, respectively, while the Ta_2_O_5_ film has refractive indices of 1.637, 1.449, and 1.589 at a wavelength of 633 nm [33]. As shown in the top-left corner of the figure, the resonance angle of the coupler is equal to 60° at a wavelength of 632.8 nm and results in a reflectance coefficient *R_pp_* ≤ 0.1.

The albumin-containing sample can be described as $S=M\times S^{\prime }$, where M is the Mueller matrix of the sample, and *S′* and *S* are the Stokes vector of the input light and output light, respectively. The use of four polarization input lights, namely 0°, 45°, 90°, and one right-hand input light, yields a sufficient number of equations to determine the Mueller matrix of sensor *M*. The Stokes vectors of those input lights are given as follows: $S_{0^{{}^{\circ}}}^{\prime }=[{\begin{array}{cccc} 1 & 1 & 0 & 0] \end{array}}^{T},$ $S_{45^{{}^{\circ}}}^{\prime }=[{\begin{array}{cccc} 1 & 0 & 1 & 0] \end{array}}^{T},$ $S_{90^{{}^{\circ}}}^{\prime }=[{\begin{array}{cccc} 1 & -1 & 0 & 0] \end{array}}^{T},$ and $S_{R}^{\prime }=[{\begin{array}{cccc} 1 & 0 & 0 & 1] \end{array}}^{T}$. The Mueller matrix of the albumin-containing sample is then given by

(1) $$M=\frac{1}{2}\left[\begin{array}{cccc} S_{0^{{}^{\circ}}}(0)+S_{90^{{}^{\circ}}}(0) & S_{0^{{}^{\circ}}}(0)-S_{90^{{}^{\circ}}}(0) & 2S_{45^{{}^{\circ}}}(0)-\left[S_{0^{{}^{\circ}}}(0)+S_{90^{{}^{\circ}}}(0)\right] & 2S_{R}(0)-\left[S_{0^{{}^{\circ}}}(0)+S_{90^{{}^{\circ}}}(0)\right]\\ S_{0^{{}^{\circ}}}(1)+S_{90^{{}^{\circ}}}(1) & S_{0^{{}^{\circ}}}(1)-S_{90^{{}^{\circ}}}(1) & 2S_{45^{{}^{\circ}}}(1)-\left[S_{0^{{}^{\circ}}}(1)+S_{90^{{}^{\circ}}}(1)\right] & 2S_{R}(1)-\left[S_{0^{{}^{\circ}}}(1)+S_{90^{{}^{\circ}}}(1)\right]\\ S_{0^{{}^{\circ}}}(2)+S_{90^{{}^{\circ}}}(2) & S_{0^{{}^{\circ}}}(2)-S_{90^{{}^{\circ}}}(2) & 2S_{45^{{}^{\circ}}}(2)-\left[S_{0^{{}^{\circ}}}(2)+S_{90^{{}^{\circ}}}(2)\right] & 2S_{R}(2)-\left[S_{0^{{}^{\circ}}}(2)+S_{90^{{}^{\circ}}}(2)\right]\\ S_{0^{{}^{\circ}}}(3)+S_{90^{{}^{\circ}}}(3) & S_{0^{{}^{\circ}}}(3)-S_{90^{{}^{\circ}}}(3) & 2S_{45^{{}^{\circ}}}(3)-\left[S_{0^{{}^{\circ}}}(3)+S_{90^{{}^{\circ}}}(3)\right] & 2S_{R}(3)-\left[S_{0^{{}^{\circ}}}(3)+S_{90^{{}^{\circ}}}(3)\right] \end{array}\right],$$

where $S_{0^{{}^{\circ}}},S_{45^{{}^{\circ}}},S_{90^{{}^{\circ}}}$, and $S_{R}$ are the output Stokes parameters corresponding to input lights with 0°, 45°, 90°, and right-hand circular polarization states, respectively. The differential Mueller matrix of albumin-containing samples is obtained as [32]

(2) m=v×(ln(λ)z)×v−1=[m11m12m13m14m21m22m23m24m31m32m33m34m41m42m43m44],

where *ν* and *λ* are the eigenvalues and eigenvectors of the Mueller matrix, *M*, respectively, and z is the axis of the coordinate system. When performing the differential Mueller matrix calculation in reflectance mode, the strong backscattering yields negative eigenvalues of the Mueller matrix, making it unphysical, and this primarily arises due to the contribution of the helicity-flipped backscattered light [32]. Nonetheless, the differential calculation can still be applied for weak scattering media in the reflectance configuration [25] or an extended differential Mueller matrix, which takes into account the sign convention [21]. In this study, the SPR prism coupler was employed to create totally internal reflectance, thus minimizing scattering. Notably, the SPR prism coupler also enhanced the performance of the detection results.

The differential Mueller matrix of an albumin-containing sample with CB/CD properties can be expressed as [22,34]

(3) $$m^{\prime }=\frac{1}{d}\left[\begin{array}{cccc} \ln\left[\left(1-R^{2}\right)\right] & 0 & 0 & \ln\left(\frac{1+R}{1-R}\right)+\kappa_{v}^{\prime }\\ 0 & \ln\left[\left(1-R^{2}\right)\right]-\kappa_{iq}^{\prime } & 2\gamma +\eta_{v}^{\prime } & 0\\ 0 & -2\gamma +\eta_{v}^{\prime } & \ln\left[\left(1-R^{2}\right)\right]-\kappa_{iu}^{\prime } & 0\\ \ln\left(\frac{1+R}{1-R}\right)-\kappa_{v}^{\prime } & 0 & 0 & \ln\left[\left(1-R^{2}\right)\right]-\kappa_{iv}^{\prime } \end{array}\right],$$

where *d* is the sample thickness, $\kappa_{iq,iu,iv}^{\prime }$ is the diagonal depolarization,$\kappa_{v}^{\prime }$ is the anomalous dichroism, *γ* is the optical rotation angle of CB, and *R* is the CD properties. By equating Equations (2) and (3), the optical rotation angle *γ* and circular dichroism *R* can be obtained respectively as [22]

(4) $$\gamma =\frac{m_{23}-m_{32}}{4},\;0\leq \gamma \leq 180^{{}^{\circ}}$$

(5) $$R=\frac{\exp\left(\frac{m_{14}+m_{41}}{2}\right)-1}{\exp\left(\frac{m_{14}+m_{41}}{2}\right)+1}$$

Finally, the glucose concentration of the albumin-containing samples can be obtained as [35]

(6) $$C=\frac{\gamma }{{\left[\gamma \right]}_{\lambda }^{}l},$$

where ${\left[\gamma \right]}_{633}=45.23$ deg/(dm g/mL) is the unique specific rotation angle of a particular molecule, and *l* is the path length and is equal to the double active layer thickness of the SPR sensor. CB is the difference in refraction of the right and left circularly polarized lights. The optical rotation angle *γ* describes the rotation of the plane of polarized light traversing a CB medium (albumin-containing glucose sample). The different glucose concentrations are associated with different optical rotation angles. As shown in Equation (6), the optical rotation angle *γ* increases linearly with the glucose concentration C. Furthermore, the circular dichroism R describes the difference in absorption of the right and left circular polarization lights caused by the difference in concentration of albumin protein. Thus, extracting *γ* and R enables the detection of glucose and albumin concentration in human blood plasma.

## 3. Experimental Setup and Results

**Figure 2:**
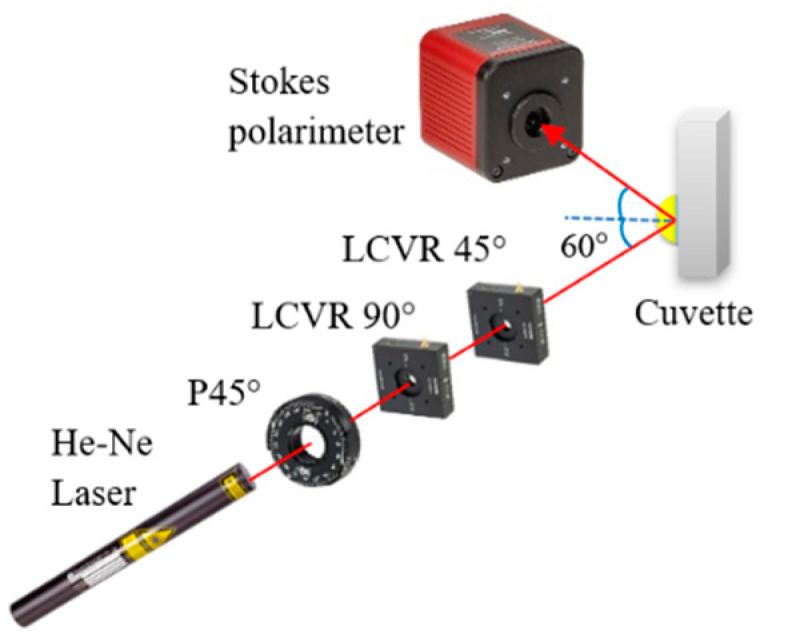
Schematic illustration of dual-retarder Mueller polarimetry system.

Figure 2 presents a schematic illustration of the proposed dual-retarder Mueller polarimetry system consisting mainly of a PSG and a commercial Stokes polarimeter (PAX1000VIS, Thorlabs Inc., Newton, NJ, USA) with an accuracy of ±25°. As shown, the PSG comprises a He–Ne laser (633 nm, 1135P, Lumentum Operations LLC, San Jose, CA, USA), a polarizer (GTH5M, Thorlabs Inc.) with the principal angle adjusted to 45°, and two LCVRs (LCC2415VIS/M, Thorlabs Inc.) with slow axis angles of 90° and 45°. The Stokes vectors of the light passing from the PSG are obtained as

(7) $$S=\mathrm{LCVR}(\delta_{2},45^{{}^{\circ}})\mathrm{LCVR}(\delta_{1},90^{{}^{\circ}})S_{in}^{\prime },$$

Thus,

(8) $$\left[\begin{array}{c} 1\\ -\sin\delta_{1}\sin\delta_{2}\\ \cos\delta_{1}\\ \cos\delta_{2}\sin\delta_{1} \end{array}\right]=\left[\begin{array}{cccc} 1 & 0 & 0 & 0\\ 0 & \cos\delta_{2} & 0 & -\sin\delta_{2}\\ 0 & 0 & 1 & 0\\ 0 & \sin\delta_{2} & 0 & \cos\delta_{2} \end{array}\right]\left[\begin{array}{cccc} 1 & 0 & 0 & 0\\ 0 & 1 & 0 & 0\\ 0 & 0 & \cos\delta_{1} & -\sin\delta_{1}\\ 0 & 0 & \sin\delta_{1} & \cos\delta_{2} \end{array}\right]\left[\begin{array}{c} 1\\ 0\\ 1\\ 0 \end{array}\right],$$

where *δ*_1_ and *δ*_2_ are the adjustable phase retardations of the two LCVRs, respectively. The output polarization states of light generated by the PSG by setting a differential set of values of *δ*_1_ and *δ*_2_ are shown in Table 1. When performing the calibration, the first LCVR was adjusted to the principal angle of 90°. The output light will be vertical and 45° at the phase retardation angles of 90°, and 0°, respectively. The second LCVR was adjusted to the principal angle of 45°, and the phase retardances of the two LCVRs were set as Table 1 to generate three linear polarization lights (0°, 45°, and 90°) and one circular polarization light (right-hand). We should note that the calibration process for the LCVR system is much easier than that of the EO system proposed in [31,32]. Furthermore, the time taken for one circle of scanning was set equal to 3 s, and the measured results of the Mueller matrix of air, the half-wave plate, and mirror are shown in Table 2. As shown, the elements of the air matrix had a maximum error of just 10^−3^, and for the half-wave plate and mirror, the matrix elements had a maximum error of 10^−2^, comparable with the accuracy and speed of the EO system proposed in [31,32]. Notably, the cost of the LCVR system is four times lower than that of the EO system.

When performing the experiments, the incident angle of the laser light was set equal to 60°. It should be noted that this is the SPR prism coupler resonance angle. In addition, the sample solution was stored in quartz cuvettes with dimensions of 10 × 10 × 1 mm^3^. Before attaching the coupler to the cuvettes, a small 6 mm-diameter hole was drilled on the cuvettes to secure that sample’s contact directly with the half-ball lens flat surface. Thus, the effect of the cuvette’s material to the measurement results can be neglected.

### 3.1. Albumin Protein Detection in Glucose Tissue Phantom Solution with 2% Lipofundin

**Figure 3:**
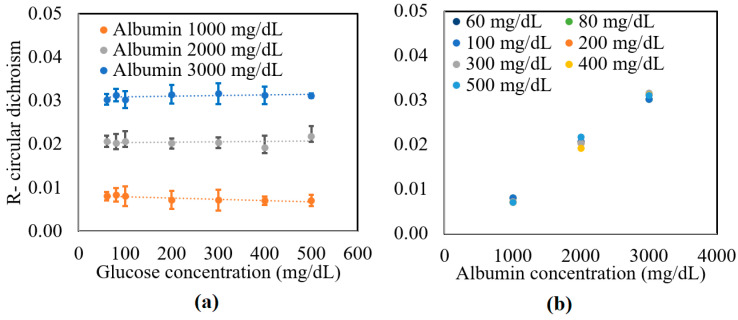
Experimental results for variation in *R* with: (**a**) glucose concentration as a function of albumin concentration, and (**b**) albumin concentration as a function of glucose concentration.

The preparation method of samples is described in detail in [32]. In brief, deionized water (DI), D-glucose (Merck Ltd., Darmstadt, Germany), and 2% lipofundin (lipofundin MCT/LC1 20%, B|Braun) were mixed with an appropriate ratio. The concentrations of the glucose sample were 0~500 mg/dL in 100 mg/dL increments. Additional glucose tissue phantom samples with concentrations of 60 and 80 mg/dL were also prepared to simulate a small glucose concentration situation. The samples were mixed with three different bovine serum albumin (Sigma Aldrich, Darmstadt, Germany) concentrations, namely 1000, 2000, and 3000 mg/dL. Figure 3 shows the experimental results obtained for the circular dichroism (*R*) properties of the various samples. As shown in Figure 3a, the circular dichroism remained unchanged as the glucose concentration increased for all three values of the albumin concentration. However, for a constant glucose concentration, the *R* value increased from 0.01 to 0.03 as the albumin concentration increased from 1000 to 3000 mg/dL. Figure 3b confirms that for all values of the glucose concentration, the circular dichroism increased linearly with albumin concentration. The average standard deviation of the measured values of *R* over four repeated tests was found to be 1.6 × 10^−2^.

**Figure 4:**
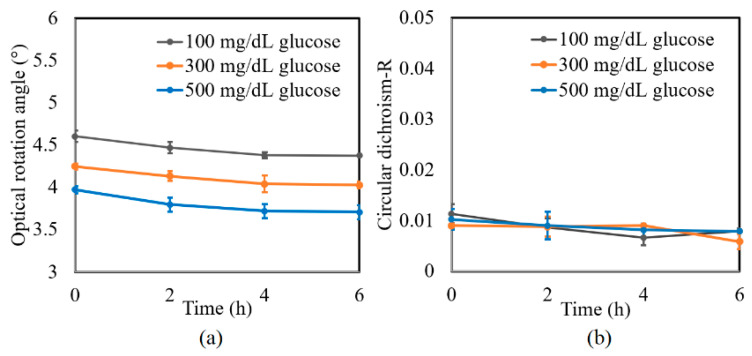
Experimental results for variation in: (**a**) *γ* and (**b**) *R* of glucose tissue phantom solutions mixed with 1000 mg/dL of albumin over time interval of 6 h.

**Figure 5:**
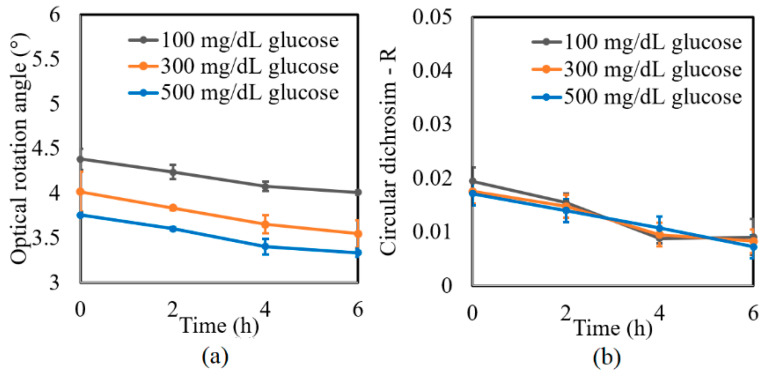
Experimental results for variation in: (**a**) *γ* and (**b**) *R* of glucose tissue phantom solutions mixed with 2000 mg/dL of albumin over time interval of 6 h.

**Figure 6:**
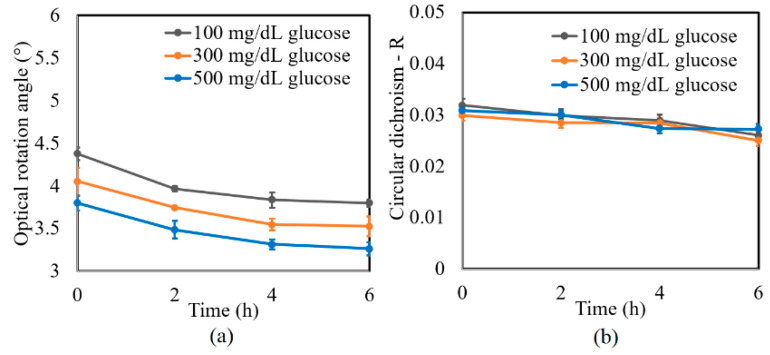
Experimental results for variation in: (**a**) *γ* and (**b**) *R* of glucose tissue phantom solutions mixed with 3000 mg/dL of albumin over time interval of 6 h.

A further series of experiments was performed to investigate the protein glycation effect of albumin by measuring the variation in the optical rotation angle and circular dichroism of glucose tissue phantom solutions with concentrations of 100, 300, and 500 mg/dL, respectively, over a period of six hours. Each sample was mixed with three different albumin concentrations, namely 1000, 2000, and 3000 mg/dL. The corresponding results are presented in Figure 4, Figure 5 and Figure 6, respectively. For all three albumin concentrations, the optical rotation angle of the glucose samples decreased rapidly over the first two hours and then continued to decrease more slowly over the remaining four hours. The CD also reduced continuously over the considered time period. Furthermore, the sample with 2000 mg/dL of albumin showed a higher reduction in CD than the samples with 1000 and 3000 mg/dL of albumin. It is suspected that for glucose concentrations over the range of 0–500 mg/dL, the protein glycation rate is highest at an albumin concentration of 2000 mg/dL. Overall, the results confirmed that in the presence of albumin, protein glycation occurs, which results in a lowering of the sugar content in the solution [8], as well as reductions in the extracted values of the optical rotation angle and circular dichroism accordingly. The average standard deviations of the extracted values of *γ* and *R* over four repeated tests were determined to be 7.9 × 10^−2^° and 2.4 × 10^−3^, respectively.

### 3.2. Glucose Concentration Detection in Albumin-Containing Media

**Figure 7:**
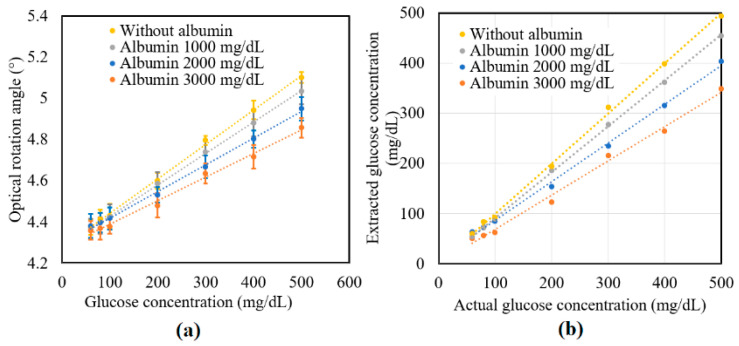
Experimental results for variation in: (**a**) *γ* and (**b**) *R* of glucose tissue phantom solutions mixed with 3000 mg/dL of albumin over time interval of 6 h.

Figure 7a shows the experimental results obtained for the variation in *γ* of the tissue phantom solutions with the glucose concentration given the addition of albumin in concentrations of 0, 1000, 2000, and 3000 mg/dL. For each of the albumin concentrations, *γ* increased linearly with glucose concentration over the considered measurement range. However, for a constant glucose concentration, *γ* decreased with albumin concentration due to the glycoxidation effect. The standard deviation of the measured values of *γ* over four repeated tests was found to be 5.2 × 10^−2^.

Figure 7b compares the extracted glucose concentrations obtained from Equation (6) with the known glucose concentrations of the various samples. Table 3 shows the extracted values of the glucose concentrations for the samples with no albumin addition. As shown, the maximum extraction error was 9.36% for the sample with a glucose concentration of 100 mg/dL. Moreover, the average error value and percentage were equal to 5.74 mg/dL and 3.34%, respectively. The accuracy of the extraction results was thus in good agreement with the value of 10 mg/dL reported in a previous study [32]. Table 4 shows the extraction results obtained for the glucose samples with different albumin concentrations. As described earlier, the glucose concentration reduced with albumin concentration due to the protein glycoxidation effect. Moreover, the extent of the reduction in the sugar level increased with glucose concentration. The protein glycation rate was faster with the higher albumin concentration. In the future, the high protein glycation samples consisting of both albumin (with actual concentration in human blood plasma—4000 mg/dL) and globulin proteins will be studied. Furthermore, a clinical study will be performed on volunteers including patients with diabetes to confirm the practical feasibility of the proposed technique for NI glucose sensing.

## 4. Conclusions

This study presented an enhanced CD/CB measurement technique based on a dual-retarder Mueller matrix polarimetry system and a gold-based SPR prism coupler in the reflectance configuration. The validity of the proposed method was demonstrated by measuring the circular dichroism (*R*) and optical rotation angle (*γ*) of glucose tissue phantom solutions with different glucose and albumin concentrations. The results showed that the circular dichroism increased with both the glucose concentration and albumin concentration. The standard deviation of the measured circular dichroism over four repeated tests was shown to be approximately 1.6 × 10^−2^. It was additionally shown that the optical rotation angle increased linearly with the glucose concentration. Notably, the results showed that the proposed technique was able to detect the reduction in the glucose concentration caused by protein glycation. The feasibility of the proposed system was demonstrated by comparing the extracted values of the glucose concentration with the known glucose concentrations of the corresponding samples. The results showed that the proposed technique was able to detect glucose with an average error of 5.74 mg/dL over the glucose concentration range of 60~500 mg/dL. Overall, the results confirmed that the proposed technique provides a promising tool for detecting the glucose concentration in real-world biological samples containing albumin, intralipid, and D-Glucose.

## Figures and Tables

**Table 1:** Output polarization states generated by dual-retarder Muller polarimetry system.

Phase Retardation of LCVR State of Polarization		State of Polarization	Stokes Vectors
LCVR 90°	LCVR 45°		
90°	270°	0°	${\left[\begin{array}{cccc} 1 & 1 & 0 & 0 \end{array}\right]}^{T}$
0°	0°	45°	${\left[\begin{array}{cccc} 1 & 0 & 1 & 0 \end{array}\right]}^{T}$
90°	90°	90°	${\left[\begin{array}{cccc} 1 & -1 & 0 & 0 \end{array}\right]}^{T}$
90°	180°	R-	${\left[\begin{array}{cccc} 1 & 0 & 0 & 1 \end{array}\right]}^{T}$

**Table 2:** Measured Mueller matrices of standard optical samples.

Air	Half-Wave Plate	Mirror
$\left[\begin{array}{cccc} 1 & 0 & 0 & 0\\ 0 & 1 & 0 & -0.0010\\ -0.0055 & -0.0045 & 1 & 0.0055\\ 0 & 0 & -0.0010 & 0.9998 \end{array}\right]$	$\left[\begin{array}{cccc} 1 & 0 & 0 & 0\\ 0 & 1 & -0.0200 & 0\\ 0.0200 & 0 & -0.9900 & 0.0173\\ 0.0009 & -0.0209 & -0.0409 & -1 \end{array}\right]$	$\left[\begin{array}{cccc} 1 & 0 & 0 & 0\\ 0 & 1 & -0.0400 & 0.0100\\ 0 & -0.0300 & -1 & 0.0150\\ 0 & 0.0100 & 0.0736 & -1 \end{array}\right]$

**Table 3:** Extraction errors for glucose concentration of tissue phantom solutions without albumin.

Actual glucose concentration (mg/dL)	60	80	100	200	300	400	500
Extracted glucose concentration (mg/dL)	58	82	91	193	311	398	492
Error value (mg/dL)	2	2	9	7	11	2	8
Error percentage	2.78%	2.19%	9.36%	3.44%	3.51%	0.59%	1.53%

**Table 4:** Extraction values of glucose concentration for tissue phantom solutions with different albumin concentrations.

Actual glucose concentration (mg/dL)		60	80	100	200	300	400	500
Extracted glucose concentration with albumin	1000 mg/dL	52	70	88	184	276	361	453
	2000 mg/dL	62	72	84	152	233	314	402
	3000 mg/dL	49	55	61	122	214	262	347

## Data Availability

Data available on request.
